# Chronic, Battery‐Free, Fully Implantable Multimodal Spinal Cord Stimulator for Pain Modulation in Small Animal Models

**DOI:** 10.1002/advs.202415963

**Published:** 2025-04-04

**Authors:** Allie J. Widman, Taron Bashar, Alex Burton, David Marshall Clausen, Prashant Gupta, Drew K. Wolf, Jakayla Folarin‐Hines, Maria Payne, John A Rogers, Kathleen W. Meacham, Robert W. Gereau, Philipp Gutruf

**Affiliations:** ^1^ Department of Anesthesiology Washington University School of Medicine St. Louis MO 63110 USA; ^2^ Washington University Pain Center Washington University School of Medicine St. Louis MO 63110 USA; ^3^ Department of Biomedical Engineering University of Arizona Tucson AZ 85721 USA; ^4^ Department of Biomedical Engineering Northwestern University Evanston IL 60208 USA; ^5^ Department of Neuroscience Washington University St. Louis MO 63110 USA; ^6^ Department of Biomedical Engineering Washington University St. Louis MO 63110 USA; ^7^ Department of Electrical and Computer Engineering University of Arizona Tucson AZ 85721 USA; ^8^ Bio5 Institute University of Arizona Tucson AZ 85721 USA; ^9^ Neroscience GIDP University of Arizona Tucson AZ 85721 USA

**Keywords:** chronic pain, neuromodulation, pain modulation, preclinical models, spinal cord stimulator, wireless implants

## Abstract

Spinal cord stimulation (SCS) for chronic pain management is an invasive therapy involving surgical implantation of electrodes into spinal epidural space. While the clinical value and mechanistic action of the therapy is debated considerably in recent years, preclinical chronic studies employing rodent models can provide invaluable insights regarding the balance between efficacy and complications as well as mechanistic understanding of SCS therapy. However, current rodent compatible devices require tethered power delivery or bulky batteries, severely limiting the ability to probe long‐term efficacy of SCS therapy. This work introduces a tether‐free, small‐footprint, fully implantable, battery‐free SCS device compatible with rodent models, capable of delivering electrical stimulation to the spinal cord at a wide range of frequency, amplitude, and period via wireless communication adjustable on‐demand without direct interaction with the animal. The presented device features capabilities of clinical SCS devices, with materials and processes amendable to scalable fabrication at a cost suitable for one‐time use enabling high N studies. In this proof of concept, the implantable device serves to assess therapeutic efficacy of various clinically relevant SCS paradigms in alleviating neuropathic pain. This technology offers chronic stability and the potential to serve as the foundation for future research into the development of SCS therapeutic systems.

## Graphical abstract

This work introduces wireless‐powered fully implantable spinal cord stimulation (SCS) in freely moving animal models, supporting diverse ethological paradigms. kHz stimulation with real‐time digital programming offers versatile, dynamically optimized therapy. Scalable fabrication ensures high N studies and seamless translation. Demonstrations show natural movement, fast recovery, and that high‐frequency SCS provides sustained pain relief over 24 h, unlike conventional SCS.

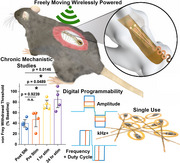

## 1 Introduction

The rapidly rising global prevalence of chronic pain, which is one of the top three causes of disability, in conjunction with the current opioid epidemic has presented an urgent need for the development of effective non‐opioid pain remediation strategies.^[^ ^1^, ^2^, ^3^ ^]^ Exploiting the landmark gate control theory of nociception reported by Melzack and Wall in 1965, Shelay and colleagues laid the foundation for spinal cord stimulation (SCS) for chronic pain treatment in 1967.^[^ ^4^, ^5^ ^]^ Since its inception more than 50 years ago, SCS has established itself as a promising non‐opioid clinical intervention alternative for people burdened with chronic pain.^[^ ^6^, ^7^ ^]^ However, owing to the invasive surgical procedure involved with SCS therapy as well as highly unpredictable and variable clinical outcomes, SCS is usually employed as a last‐resort treatment only after all other viable clinical interventions are exhausted.^[^ ^6^, ^8^ ^]^

The physiological rationale for the conventional SCS paradigm, which involves tonic low frequency (typically 30–100 Hz) electrical stimulation of large fibers (Aβ fibers), is based on the gate control theory that describes how painful sensations (signaling via small C and Aδ fibers) can be overridden and reduced by non‐painful sensations (signaling via large Aβ fibers).^[^ ^8^, ^9^, ^10^ ^]^ Under the conventional SCS paradigm, stimulation of Aβ fibers antidromically inhibits pain, but orthodromically generates paresthesia which can be uncomfortable at higher stimulation amplitude, thereby restricting its therapeutic efficacy for pain remediation.^[^ ^9^, ^11^, ^12^ ^]^ On the other hand, extensive developments over the past 10 years have resulted in newer stimulation modalities, such as High Frequency (HF), burst stimulation, and differentially targeted multiplex (DTM), which produce significant pain relief without causing the unpleasant paresthesia.^[^ ^13^, ^14^, ^15^ ^]^ However, the mechanism of action of newer stimulation modalities cannot be explained solely based on the direct stimulation of large fibers, suggesting more complex mechanisms at play that are yet to be elucidated.^[^ ^11^, ^16^, ^17^ ^]^ Moreover, the field has limited evidence and consensus on what neural mechanisms contribute to analgesic spinal stimulation, particularly when comparing different SCS modalities.^[^ ^18^, ^19^ ^]^ This knowledge gap in mechanistic understanding is considered one of the primary bottlenecks that drastically affects clinical outcomes.^[^ ^8^, ^20^ ^]^

Rodent models have been employed extensively in the biomedical field as they allow for in‐depth investigation of pathological and physiological events.^[^ ^21^, ^22^ ^]^ However, investigation of analgesic mechanisms of different SCS paradigms using rodent models is constrained by current device limitations. In typical setups, an animal is tethered to an external stimulator at a connector on the head of the animal and internalized wires travel back to the lumbar spinal cord.^[^ ^23^, ^24^ ^]^ While commonly utilized, the wires and connectors are often damaged by the animal or housing arrangements posing an obstacle for long‐term continuous stimulation experiments. This effectively limits studies to short‐term or low N paradigms that avoid naturalistic environments and motion‐intense protocols with many sessions.^[^ ^25^, ^26^ ^]^ Battery‐powered systems are bulky and continuous stimulation in complex behavior setups is hindered by battery recharging and frequent experimenter interaction.^[^ ^25^, ^26^ ^]^ To solve these issues, we have created a wireless, battery‐free spinal cord stimulator that is programmable and able to perform a variety of patterns to effectively study chronic stimulation paradigms without manual interaction with the subject to enable automated chronic studies that make statistically relevant experimental protocols and paradigms feasible.

## 2 Results

### 2.1 Wireless SCS Device Overview

**Figure 1:**
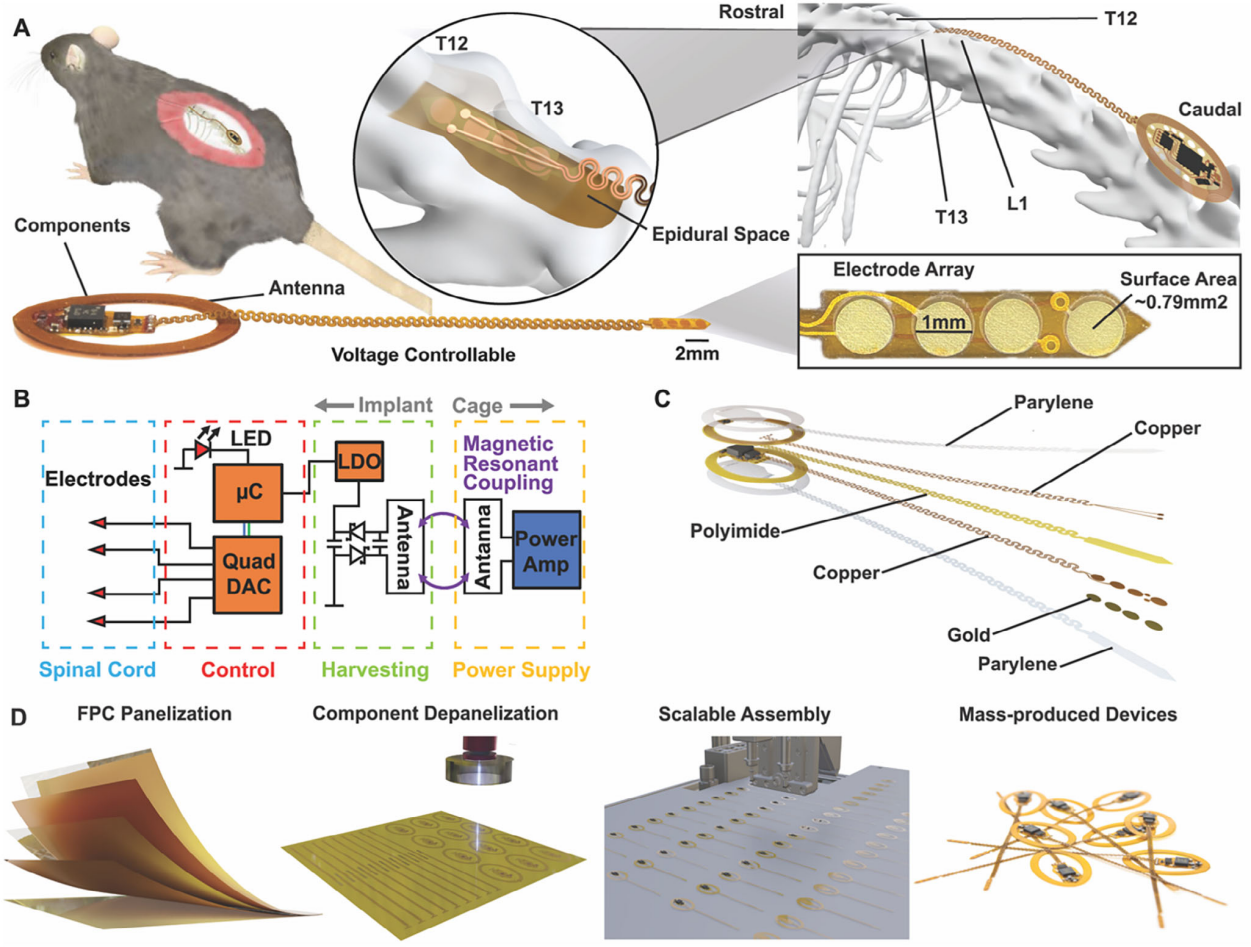
Design and development of wireless, fully implantable spinal stimulation device A) Overview of spinal stimulation device with detailed display of implantation site showing anatomical landmarks B) Block diagram displaying functional circuit C) Exploded view of the constituent layers of device D) Fabrication process optimized for repeatability and scalability.

The modular design of the flexible circuit for the multimodal spinal cord stimulator (M‐SCS) integrates distinct aspects of the device into a single system (**Figure** **1**A), capable of wireless communication and voltage‐controlled neurostimulation within its minimal physical footprint. The device antenna limits the spacing between the seven turns to 1 mm to help minimize this physical footprint, where the device itself is designed on a dual‐sided, flexible printed circuit board (PCB) with a thickness of 104 µm, incorporating the insulating polyimide layer and conductive copper traces. The thin, flexible profile of the M‐SCS allows for adjustable localization along the spinal cord during implantation and enables better device integration, while the minimal footprint further improves the recovery time for the subject post‐implantation. Efficient wireless power transfer (WPT) to the device is achieved through magnetic resonan coupling, by tuning the antenna on the device body that is coupled with a monolithically integrated resonator to 13.56 MHz.^[^ ^27^ ^]^ Two system voltages are created with daisy‐chained low‐dropout regulator (LDO) creating 5 and 2.8 V respectively. The digital system is centered around the microcontroller (Atmel; ATTINY84A), powered at 2.8 V) as shown in the schematic (Figure 1B). The lowered operational voltage is selected to reduce the power consumption of the digital components. The microcontroller communicates with the quad‐channel digital‐to‐analog converter (DAC) (Maxim Integrated; MAX5715) through the serial peripheral interface (SPI) serial communication protocol, which allows for control over the DAC channels. This allows for control over the output voltage generated by the DAC which is powered using a 5 V signal to enable higher stimulation voltage compliance with a biphasic stimulation that allows for charge balancing across the electrodes, thereby improving stimulation response and reducing tissue damage and corrosion at the electrode‐tissue interface during chronic stimulation.^[^ ^27^, ^28^ ^]^ A multilayer flexible stimulation interface (Figure 1C), which houses the four gold‐plated, circular electrodes, each with a diameter of 1 mm and a contact area of 0.79 mm^2^ (Figure 1A). Gold was selected as the electrode material to enable low‐cost fabrication without additional electrode coating steps that are needed with standard stimulation electrode materials such as platinum‐iridium. The flexible serpentine connects the four‐channel electrode array to the primary device module, enabling strain isolation of the electrode array.^[^ ^29^, ^30^, ^31^ ^]^ This feature allows for a greater range of motion during surgery for precise probe placement in the epidural space while maintaining electrical connectivity and structural stability.^[^ ^32^, ^33^ ^]^

For chronic stability and biocompatibility, the devices are coated with a conformal layer of Parylene‐C as an encapsulant, providing a moisture barrier.^[^ ^34^ ^]^ The fully assembled devices are coated with Ecoflex (Smooth‐On; Silicone), which is a soft elastomer to provide modulus matching with the surrounding tissues. This results in lower fibrotic response and sustains the overall soft mechanics of the device, to retain its conformability.^[^ ^27^, ^33^, ^35^ ^]^ The device architecture is highly optimized for repeatable and scalable manufacturing using commercially available surface‐mounted components (Figure 1D) and a panelized fabrication process.^[^ ^27^, ^36^ ^]^ The panelization process for the Flexible Printed Circuit (FPC) illustrates a systematic approach, facilitating efficient laser milling (depanelization) and the potential for mass assembly through a pick‐and‐place machine (Figure 1D). This, coupled with the optimized design incorporating commercially available surface‐mounted components, ensures repeatability and scalability. Moreover, compatibility with reflow soldering further enables scaled device assembly, contributing to the broad dissemination of devices beyond the academic community.

### 2.2 Resonant Antenna Design and Characterization

**Figure 2:**
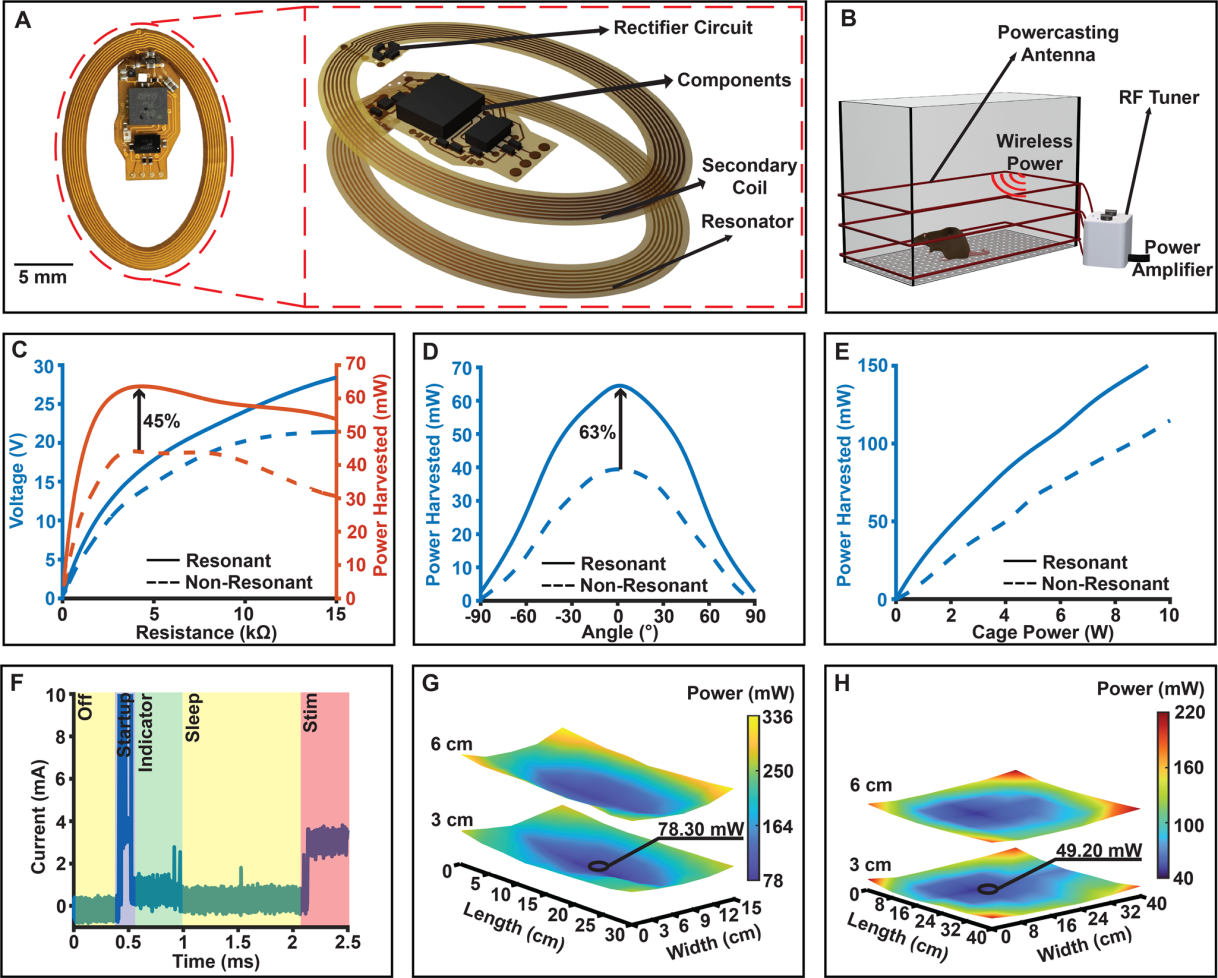
Power harvesting and distribution characteristics of the spinal stimulation device A) Resonant antenna displayed with exploded view B) Overview of the device powering in the experimental arena. C) Power harvesting capability of device with non‐resonant versus resonant antenna shown against varying load at 4 W input RF power D) Power harvesting capability of device with non‐resonant versus resonant antenna shown against varying device angle at 4 W input RF power E) Power harvesting capability of device with non‐resonant versus resonant antenna against varying RF input power F) Transient power consumption of spinal stimulation device at 5 V system voltage G) Spatial distribution of power harvested by antenna with passive resonator at 5 W input RF power for 30 cm × 15 cm cage H) Spatial distribution of power harvested by antenna with passive resonator at 8 W input RF power for 40 cm × 40 cm cage.

High voltage compliance plays a crucial role in activating excitable tissues especially when contact impedance is variable. It ensures accurate and reliable delivery of the required currents and voltages, addressing the specific needs of the targeted tissue. However, implementing such high voltages with sufficient current to drive stimulation reliably, into a traditional 2‐antenna system poses challenges due to limitations in inductance ratio and coupling between antennas.^[^ ^27^ ^]^ Especially when miniaturization is desired, fundamental limitations are imposed as smaller device footprints result in lower power transfer (Figures S1–S3, Supporting Information). To overcome these limitations, we introduce a passive resonator monolithically integrated into the device body (**Figure** **2**A). This implementation improves magnetic resonance coupling between the antennas and enhances power transfer by lowering the maximum power point of the system, enabling higher inductance of the antenna at the same device footprint, increasing efficiency. Within the experimental paradigm, the M‐SCS antennas are optimized to a 3‐turn primary (transmitter) antenna surrounding the enclosure (30 cm × 15 cm), with 2.5 cm spacing between the coil turns (Figure 2B). Here, we observe a significant increase in harvested power with the addition of the passive resonator to the device. Specifically, when the operational load of the system is matched, we observe a 45% increase in harvested power (Figure 2C). Considering the motion of small animals such as rats that often include rearing,^[^ ^36^, ^37^ ^]^ the device needs to harvest substantially more power than its peak use for continuous stimulation across a wide range of angular mismatches with the primary antenna. At the center of the enclosure, the power harvesting capability of the device with the resonator is significantly higher as the device rotates between −90° to 90° angles simulating angular mismatch during naturalistic behavior.^[^ ^36^, ^37^ ^]^ At the base position (0° to the horizontal axis), the device can harvest 63% more power compared to the non‐resonant version (Figure 2D).

Minimizing implant footprint and displacement volume is a key consideration for wireless, fully implantable devices designed for small animal models.^[^ ^27^, ^32^, ^36^ ^]^ Here, efficient wireless power transfer (WPT) at lower radio frequency (RF) powers helps retain the ability to increase RF power (Figure 2E) to enable operation in large experimental enclosures. Current consumption during maximum voltage stimulation (±5 V amplitude), the M‐SCS draws current ranging from 1 to 10 mA depending on tissue load and program sequence, (Figure 2F). Peak power drawn during device startup of ≈56 mW is buffered with on board capacitors (2.2 uF) where average power consumption of ≈20 mW during stimulation can be harvested even at low primary antenna power settings of 2 W in the center of the cage where the implant harvests the least amount of power, the harvested power is still ≈60 mW (Figure 2D–G). The maximum power limited by amplifiers for 13.56 MHz systems is 10 W enabling continuous operation in larger‐sized cages where benchtop testing reveals that devices can comfortably operate in a cage size of 40 cm × 40 cm (Figure 2H) with an input RF power of 8 W with a minimum harvested power at the center of 49.5 mW which is more than double the power needed for continuous operation.^[^ ^27^ ^]^

### 2.3 Electrode and Stimulation Control

**Figure 3:**
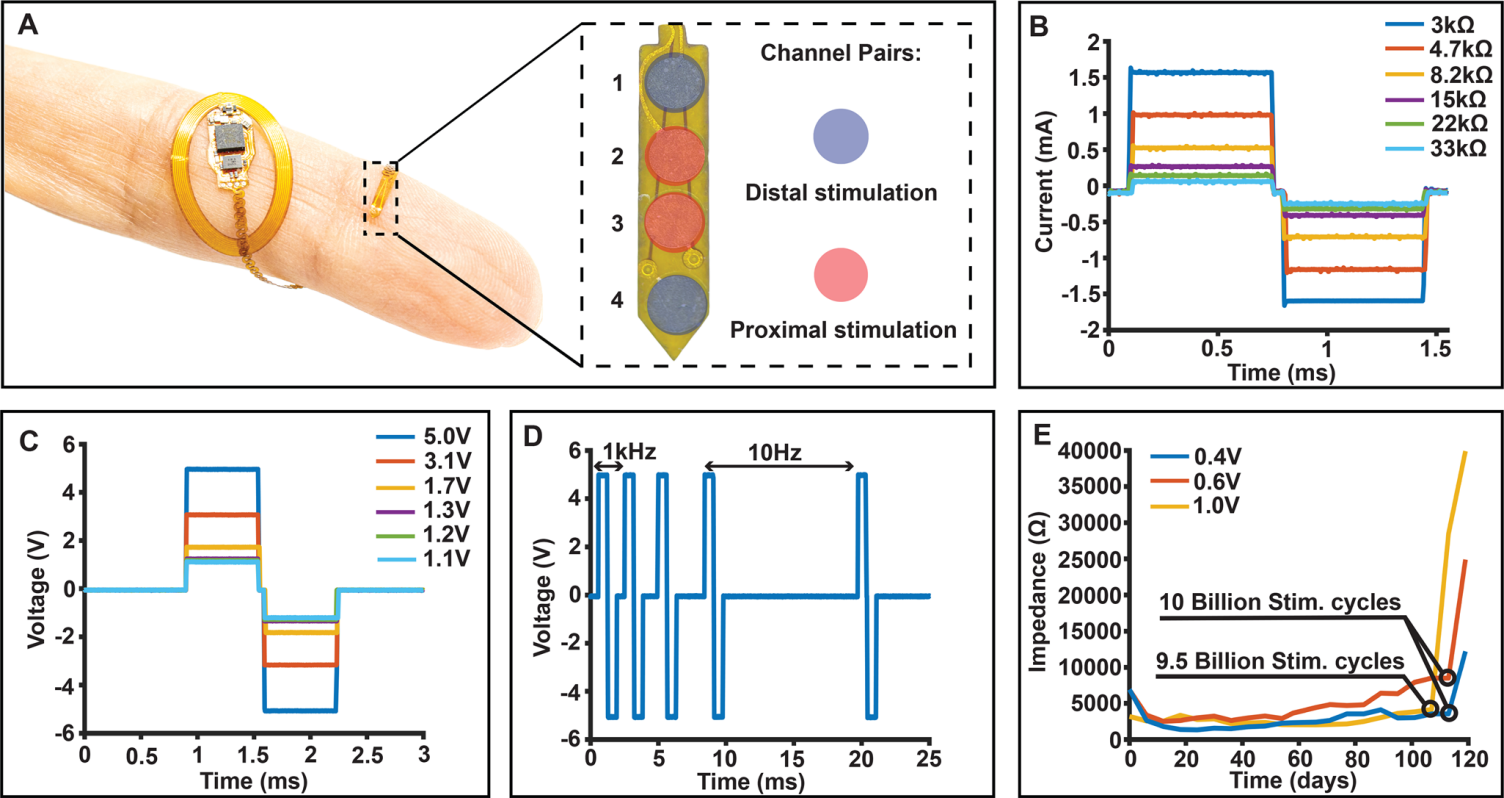
Functional capabilities and stability of the spinal stimulation device. A) Flexible device and serpentine displayed on the finger with zoomed‐in view of the electrode array and channels used for stimulation. B) Stable current delivery through electrodes against varying loads. C) Amplitude modulation capability of device. D) Period modulation capability of device. E) Chronic impedance testing displaying stability of electrodes at continuous 1 kHz stimulation.

The multi‐electrode array (**Figure** **3**A) is designed to deliver a biphasic pulse with localized current injection. Given the full digital control over the DAC which features high impedance states, any combination of electrodes can be programmed. Electrode pairs can therefore be more selective if adjacent electrodes are used or can target a larger area when electrodes at the far end are used as a stimulation pair (Figure 3A). The surface area of the electrodes, which are 1 mm in diameter, is chosen to be similar to commercial electrodes in humans when scaled by spinal cord diameter for a rat.^[^ ^38^ ^]^ During stimulation, stable voltage for biphasic stimulation is maintained across a wide range of electrode impedances, allowing for current delivery ranging from 0.1 to 1.6 mA (Figure 3B) for the tested range of impedances across the electrodes (3–33 kΩ), which is well above currents used for functional stimulation of the spinal cord.^[^ ^39^ ^]^ Communication between the microcontroller and the DAC allows for precise control over various aspects of biphasic stimulation, such as amplitude (0.1–5 V) (Figure 3C), pulse width (600–10 µs) (Figure S4, Supporting Information), and period (1 ms– 1 s) (Figure 3D). The implant can be programmed using a custom RF amplitude shift keying (ASK), where 3 bits are transmitted for channel selection, 6 bits for amplitude value, 6 bits for pulse width value, and the final 6 bits for period value.^[^ ^27^ ^]^ M‐SCS stimulation electrode interfaces are tested in PBS solution to simulate physiological conditions and evaluate stability in current delivery. PBS is used as an analog to mimic the conductivity and electrolyte composition of cerebrospinal fluid. Our electrical testing demonstrates stable current delivery up to 3.3 mA (Figure S5, Supporting Information) across any pair of electrodes under these conditions.

### 2.4 Chronic Wireless Operation

To demonstrate chronic stability, the entire device is characterized in an accelerated rate test at 37, 60, and 90 °C, and functionality is recorded every third day.^[^ ^36^ ^]^ All the devices are functioning as of day 64 and Arrhenius scaling is used to estimate the lifetime for the devices at 60–90 °C (Figure S6, Supporting Information), resulting in a theoretical lifetime of at least 264 days at 37 °C.^[^ ^40^ ^]^ To characterize the durability of the electrodes under chronic stimulation, we perform continuous stimulation of three separate electrodes at 1 kHz,^[^ ^27^, ^33^, ^36^ ^]^ with decreasing voltage levels (1, 0.6, and 0.4 V) in PBS solution (Figure 3E). The stimulation is conducted continuously at ±1 V at the electrode interface to remain just within the electrode water window. The stress test protocol of 120 days of continuous stimulation while submerged in PBS solution represents the worst‐case scenario of an experiment where continuous stimulation is required (this is unlikely the case for an in vivo experimentation).^[^ ^27^, ^41^ ^]^ Notably, impedance values decreased consistently for all voltage levels throughout the initial experiment period as the surface area of the electrode increases by electrochemical contaminant removal through stimulation and increasing surface roughness morphology change.^[^ ^42^, ^43^ ^]^ As anticipated, the 1 V stimulation electrodes exhibit the highest degradation rate and fail after 9.5 billion stimulation cycles, followed by the 0.6 and 0.4 V electrodes which both displayed electrode failure after ≈10 billion stimulation cycles (Figure 3E). The degradation mechanism is highlighted by the discoloration observed around the gold plating of the parylene and polyimide encapsulation edge of all three electrodes over the 120 day test period (Figure S7, Supporting Information) and ultimately results in disconnection of the trace to the electrode (Figure S8, Supporting Information), an expected failure mechanism observed elsewhere in literature.^[^ ^44^ ^]^ The electrode performance is more than sufficient for the intended use in small animal models where experimental periods rarely exceed 3 months which this electrode style is clearly capable of supporting. It is important to note that longer lifetimes could be accomplished with additional electrode coatings such as IR‐Pt or other state‐of‐the‐art electrodes, which however would come at a significant additional cost. Since this device is intended to be single‐use for most experimental paradigms in rodents, the demonstrated electrode configuration is likely the best choice.

### 2.5 Compatibility of Wireless SCS Device

**Figure 4:**
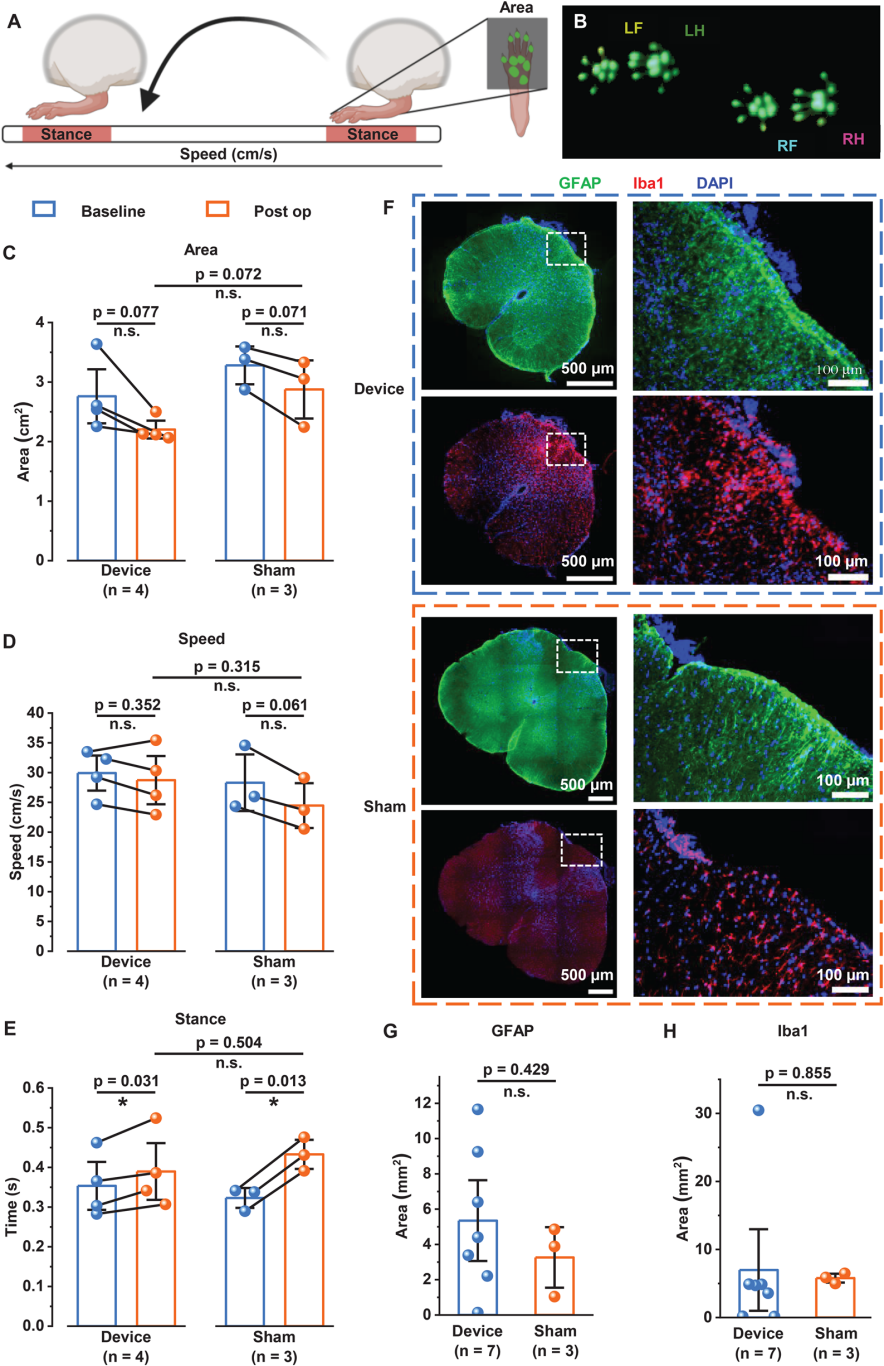
Wireless SCS device implants do not significantly alter motor function and spinal cord tissue integrity compared to sham‐operated rats. A) Schematic representation of motor function analysis on the catwalk where the hind paw print area, body speed, and hind paw stance time were measured before and after device implantation. Schematics were created with BioRender.com. B) Representative catwalk image demonstrating all paws in contact with the glass walkway (LF: left forepaw, RF: right forepaw, LH: left hindpaw, RH: right hindpaw). Pre‐ and post‐operative analysis of C) hindpaw surface area, D) body speed, and E) hindpaw stance time from a device implanted and sham rats (n ≥ 3 rats per group). Data presented as mean ± S.E.M., individual data points are depicted as filled circles and the solid line depicts the pre‐ and post‐op values for the same animal. Statistical analyses were performed via two‐sided paired two‐samples t‐test within device and sham groups and via two‐sided unpaired two‐samples t‐test for inter‐group assessment; n.s. (not significant) *p* > 0.05, ^*^ *p* < 0.05, ^**^ *p* < 0.01, ^***^ *p* < 0.001 and ^****^ *p* < 0.0001. F) Representative fluorescence images of transverse spinal cord sections from device implanted and sham rats. Spinal cord sections are co‐stained with GFAP (green) and Iba1 (red) which are biomarkers for astrocytes and microglia/macrophages respectively, along with DAPI for nucleus staining. Images on the left represent the whole spinal cord section and images on the right are the magnified image of the region marked by the white dashed box. Comparison of the total area of G) GFAP and H) Iba1 expressing cells in the dorsal region of device implanted and sham rats (n ≥ 3 rats per group). Data are presented as mean ± S.E.M. and individual data points are depicted as filled circles. Statistical analyses were performed via two‐sided unpaired two‐sample t‐test; n.s. (not significant) *p*> 0.05, ^*^ *p* < 0.05, ^**^ *p* < 0.01, ^***^ *p* < 0.001 and ^****^ *p* < 0.0001.

The SCS device implantation procedure involves the physical placement of electrodes in the epidural space, which presents a potential for damaging the spinal cord in freely moving animals and could lead to motor impairment. Accordingly, we sought to investigate the effect of implanted devices on motor function via a gait analysis system. We employ hind paw print area, body speed, and hind paw stance time, which are general parameters of gait, to assess motor function pre‐ and post‐implantation of the device (**Figure** **4**A,B).^[^ ^45^, ^46^ ^]^ Since the device is implanted in the lumbar region of the spinal cord, the motor function assessment primarily focuses on the hind paws. Although, in the device‐implanted animals, we observe a significant increase in the paw stance time and a trend of reduction (albeit statistically non‐significant) in the paw print area and the body speed pre‐ and post‐implantation (Figure 4C–E), this is also true in the case of sham‐operated animals. Moreover, statistical analyses revealed no significant differences in post‐operative values for hindpaw print area, body speed, and hindpaw stance time between the device implantation and sham surgery groups. Additionally, percent change comparisons of functional parameters among device and sham groups highlighted a significant difference only in hindpaw stance time whereas no significant difference was observed in hindpaw print area as well as body speed, further demonstrating the minimal impact of device implantation on functional recovery. It is worth noting that the device implantation surgery involves partial laminectomy along with significant removal of tissues at the implant site and the motor function assessment is performed after only 5 days of recovery from surgery. This suggests that the observed effect on motor function is attributable to the trauma suffered during the surgical procedure instead of the presence of the implanted device in the epidural space.

To further confirm that the presence of the stimulating electrode in the epidural space does not affect spinal cord integrity, we conducted histological studies on spinal cord tissue under the implanted device at 8–10 days after implantation. Owing to the fact that damage to the spinal cord triggers the proliferation of astrocytes as well as microglia,^[^ ^47^, ^48^, ^49^ ^]^ we compare the immunoreactivity level of GFAP (a biomarker for astrocytes) and Iba1 (a biomarker for microglia/macrophages) in the dorsal region of device implanted rats with those of sham to assess the device associated damage to the spinal cord (Figure 4F). We observe no statistically significant difference in the expression level of GFAP or Iba1 between device‐implanted and sham rats (Figure 4G), suggesting minimal tissue damage associated with device implantation. These results together demonstrate the compatibility of the device for long‐term implantation applications.

### 2.6 Wireless SCS Device Assisted Supra‐ and Sub‐Perception Modulation for Pain Management

**Figure 5:**
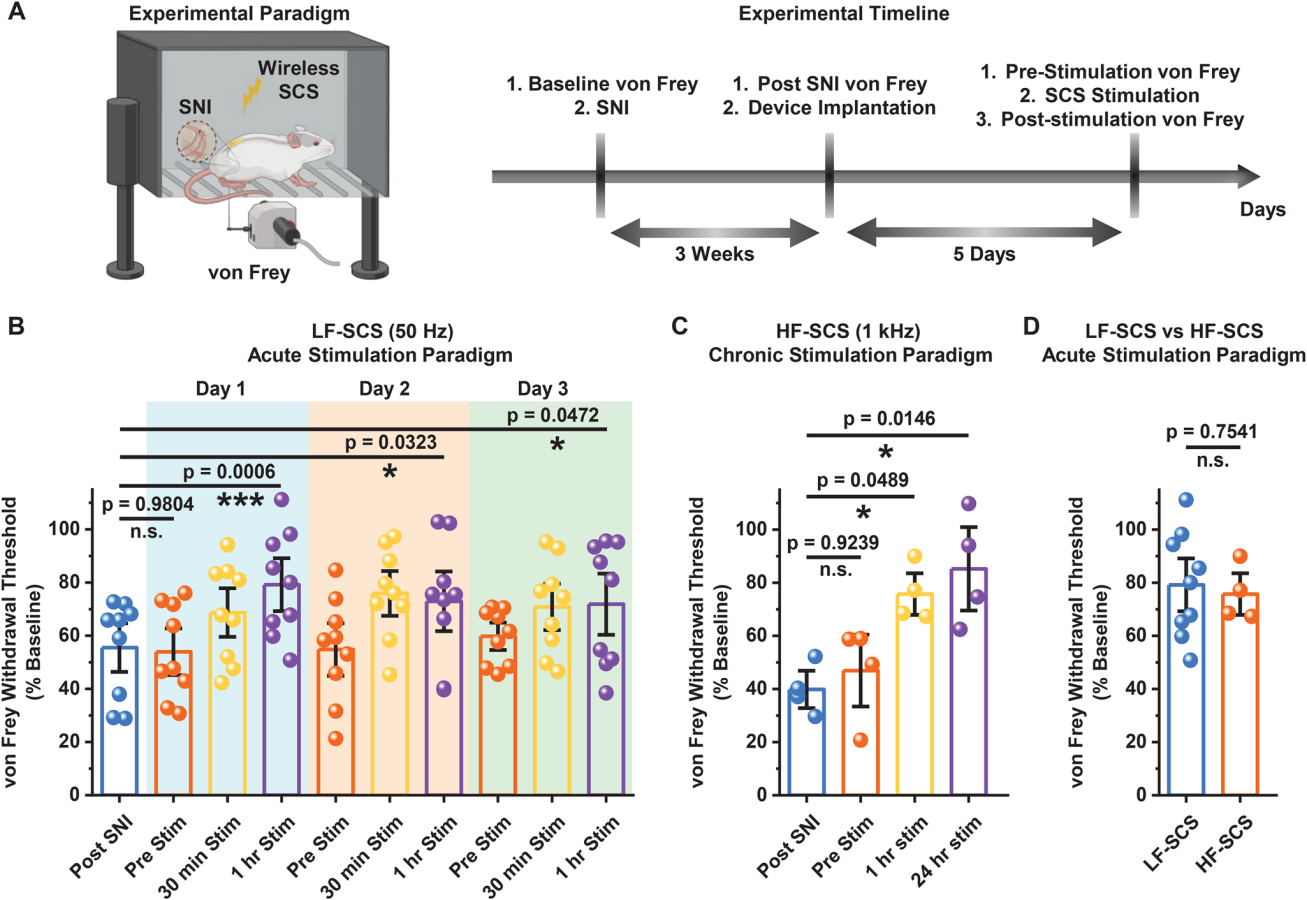
Efficacy of wireless implantable device assisted supra‐ (Low frequency) and sub‐perception (High frequency) SCS in alleviating SNI‐induced mechanical hypersensitivity. A) Schematic representation of experimental paradigm and timeline to assess the efficacy of wireless device‐assisted SCS protocols in attenuating SNI‐induced mechanical hypersensitivity. Schematics were created with BioRender.com. Paw withdrawal threshold from the rats, with both SNI‐induced hypersensitivity and implanted wireless SCS device, pre‐ and post‐ B) low‐frequency SCS under acute stimulation paradigm (30 min and 1 h stimulation) on three consecutive days (n = 9 rats) and C) high‐frequency SCS under chronic stimulation paradigm up to 24 h (n = 4 rats). B, C) Data presented as mean ± S.E.M. and individual data points are depicted as filled circles. Statistical analyses were performed via repeated measure one‐way ANOVA followed by the Tukey's post hoc test; n.s. (not significant) *p*> 0.05, ^*^ *p* < 0.05, ^**^ *p* < 0.01, ^***^ *p* < 0.001, and ^****^ *p* < 0.0001. D) Comparison of efficacy of supra‐ and sub‐perception SCS paradigm under similar stimulation duration of 1 h in relieving SNI‐induced mechanical hypersensitivity (n ≥ 4 rats per group). Data are presented as mean ± S.E.M. and individual data points are depicted as filled circles. Statistical analyses were performed via two‐sided unpaired two‐samples t‐test; n.s. (not significant) *p* > 0.05, ^*^ *p* < 0.05, ^**^ *p* < 0.01, ^***^ *p* < 0.001, and ^****^ *p* < 0.0001.

Next, we employ the developed wireless SCS device in evaluating the efficacy of various SCS modalities used for pain management including conventional (supra‐perception) and high‐frequency (sub‐perception) stimulation. We leverage the spared nerve injury (SNI) model in rats, a preclinical model that displays increased sensitivity to mechanical stimulation of the hind paw, to assess the ability of the wireless SCS device. SNI in rats produces stable hypersensitivity in the hind paw after 3 weeks, after which the wireless device is implanted. After 5 days of recovery post‐implantation surgery, the device is wirelessly triggered to investigate the SCS efficacy of various modalities to reduce the injury‐induced hypersensitivity (**Figure** **5**A). Baseline is defined by the withdrawal threshold prior to SNI surgery. The paw withdrawal thresholds for post‐SNI, pre‐stimulation, and post‐stimulation at different time points are normalized to the baseline. Prior to initiating the SCS, motor thresholds are determined (see details in the Experimental Section).

Considering conventional supra‐perception SCS results in paresthesia,^[^ ^9^, ^11^, ^12^ ^]^ we employ an acute stimulation paradigm that involves stimulation over a brief period of time. The motor thresholds are determined by leveraging the pulse width modulation capability of the device (Figure S4, Supporting Information) at a fixed amplitude of 3.3 V. We observed motor thresholds at 35–50 µs pulse widths for all animals. Under the acute stimulation paradigm, the rats are subjected to conventional SCS (LF‐SCS at 50 Hz frequency) therapy for 1 h duration for three consecutive days, and withdrawal thresholds are measured at 30 min and 1 h into stimulation (Figure S9, Supporting Information). We observe significant alleviation of mechanical hypersensitivity at the 1‐h stimulation timepoint on all three days of SCS therapy (Figure 5B). However, a significant reduction in mechanical hypersensitivity is observed after 30 min of stimulation on the first day of SCS therapy. The rats exhibit similar mechanical hypersensitivity on each day prior to initiating stimulation therapy, suggesting that there is no carry‐over effect of conventional SCS under these conditions. We observe a downward trend in the efficacy of LF‐SCS as the therapy progresses over days, although this reduction is not statistically significant. These results show a lack of carry‐over and possible reduction in efficacy over time are also observed in various clinical trials utilizing conventional SCS therapy for chronic pain management.^[^ ^6^, ^8^, ^10^ ^]^

High‐frequency SCS (HF‐SCS), also termed sub‐perception or paresthesia‐free stimulation, is also reported to provide effective therapy for pain management.^[^ ^9^, ^10^, ^11^, ^12^ ^]^ We also assess the effectiveness of HF‐SCS (1 kHz frequency) using chronic stimulation by harnessing the high‐frequency modulation capability of the developed device. In this case, we leverage the amplitude modulation capability of the device (Figure 3C) at a fixed pulse width of 100 µs to determine motor thresholds, which are observed to be 0.5–1 V for all animals in this study. We observed a significant reduction in the SNI‐induced mechanical hypersensitivity after 1 h of HF‐SCS therapy that sustained even after 24 h of chronic stimulation (Figure 5C). We observe no significant difference in the reduction of mechanical hypersensitivity between LF‐ and HF‐SCS therapy using our device. Considering both similar and contradicting observations are reported in the literature, a more systematic investigation is warranted for future studies.^[^ ^10^, ^50^ ^]^ We envision that the implantable device for small animal models presented in this work could serve as a valuable toolkit in determining the efficacy of various modalities of SCS therapy for pain modulation.

## 3 Discussion

Technological advancements in SCS devices over the past three decades have substantially transformed the field of SCS therapy for chronic pain management, as evidenced by rapidly growing number of FDA‐approved implantable SCS devices and reported successful clinical trials. Yet, the field of SCS remains divergent with competing narratives over its therapeutic efficacy and it is employed as a last‐resort clinical intervention for pain management.^[^ ^8^, ^20^, ^51^ ^]^ This stems from the limited understanding of the mechanisms of action of SCS, inconsistent clinical outcomes across various trials, and direct industry involvement in the majority of clinical trials with favorable outcomes.^[^ ^11^, ^16^, ^17^, ^52^, ^53^, ^54^ ^]^ Consequently, there is an increasing demand for industry‐independent research from neuromodulation practitioners. Preclinical studies involving rodent models could prove invaluable in moving the field of SCS forward.^[^ ^55^, ^56^ ^]^ However, current devices such as tethered systems, and bulky battery‐operated devices among others severely restrict the unhindered long‐term therapeutic efficacy investigation of SCS in rodent models.^[^ ^9^, ^23^, ^24^, ^25^ ^]^

To address these bottlenecks, a wireless, battery‐free, small footprint, fully implantable device for rodents, capable of delivering electrical stimulation to the spinal cord at a wide range of frequency, amplitude, and period that can be effortlessly adjusted on‐demand is demonstrated. The device reported in this work features the majority of the stimulation capabilities offered by most of the FDA‐approved devices.^[^ ^51^ ^]^ The devices are constructed using non‐ferromagnetic off‐the‐shelf components, speaking to not only the device's ability to undergo non‐invasive 3D medical imaging while implanted in the subject but also the ability to scale the devices for mass manufacturing.^[^ ^27^, ^32^, ^36^ ^]^ Moreover, the power harvesting capability of the device allows for the system to function indefinitely post‐implantation without the need for additional surgeries in comparison to other battery‐powered systems. The device is also equipped with a multi‐electrode array, where the 1 mm diameter electrodes^[^ ^57^ ^]^ are chosen to mimic clinical electrodes used in humans,^[^ ^58^ ^]^ allowing for current densities^[^ ^59^ ^]^ similar to those used for pain management in human subjects improving translational relevance compared to currently available electrodes designed for mapping of the rat spinal cord. These characteristics highlight the system's ability to serve as a platform for performing chronic preclinical studies, having the opportunity to closely study the effect of frequency (especially the higher frequency range), amplitude, and pulse width modulation of the electrical pulses on the analgesic effect of the stimulation.

This study further demonstrates the capability of the developed device for investigating the therapeutic efficacy of various SCS paradigms (Figure 5). The long‐term in vivo biocompatibility of the device (Figure 4) enables both acute as well as chronic stimulation paradigms. This study emphasizes glial responses (GFAP and IBA1) to device implantation, as they represent immediate and robust tissue reactions.^[^ ^60^ ^]^ Chronic tissue responses to untethered microelectrode implants show glial activation to be more pronounced and sustained than neuronal changes.^[^ ^61^ ^]^ Additionally, subdural bioelectronic implants alter glial populations without significant long‐term motor function deficits, highlighting the importance of glial activity as a sensitive marker of spinal cord response.^[^ ^62^ ^]^ While these findings justify the focus on glial markers, future studies should include neuronal markers to complement these analyses and capture potential functional and structural changes in neurons. The pulse‐width modulation as well as amplitude modulation capability of the device offers precise control in determining motor thresholds and fine‐tunability of the stimulation parameters. Successful employment of the implantable device to assess the efficacy of SCS therapy is observed by alleviating SNI‐induced mechanical hypersensitivity in a rat model via both supra‐perception (conventional LF‐SCS) and sub‐perception/paresthesia‐free (HF‐SCS) stimulation protocols. Although, the capabilities of the developed device in wirelessly delivering SCS therapy are demonstrated for short duration (up to 3 days), the absence of any tissue damage from the device along with the chronic wireless operation capability of the device (up to 120 days) suggests the possibility for studies of long term SCS therapy. This is further supported by observing the function recovery of the animal (Video S1, Supporting Information), which highlights the behavioral performance of a rat before, during, and after SCS treatment. Moreover, the wireless nature of the device allows for free movement of the animals, offering the potential for assessment of complex and social behaviors. This opens novel applications for investigating the effect of social factors on the efficacy of SCS therapeutics.^[^ ^63^ ^]^

In these experiments, a significant alleviation of mechanical hypersensitivity via both supra‐perception and sub‐perception SCS is observed, and we find no significant difference in efficacy between the two SCS protocols. Observing hind limb muscle twitches during dosing sessions further verifies that electrical stimuli reached the relevant spinal circuits (Video S2, Supporting Information), underscoring the device's robust engagement of motor pathways. These demonstrations support our central premise that targeted spinal stimulation can modulate nociceptive pathways, thereby contributing to the observed analgesic effects. Such a direct, in vivo demonstration aligns with prior chronic implantation findings, reinforcing the potential for practical pain management applications.^[^ ^64^, ^65^, ^66^ ^]^ However, the small number of subjects included here warrants further systematic investigation in future studies. The purpose of this work is to provide an advanced toolkit for preclinical animal model studies that appropriately mimics SCS devices used in clinical settings. Moreover, the ability to scale the device for mass manufacturing allows for devising unbiased statistically robust therapeutic efficacy studies with higher subject numbers in a cost‐effective manner. With these device attributes, investigations aimed at assessing the efficacy of various SCS paradigms as well as elucidating mechanisms of action are enabled. This technology platform may serve as the foundation for future research into the development of SCS therapeutic systems for rehabilitation and chronic pain management.

## 4 Experimental Section

### Device Fabrication

The devices were designed in four individual parts for a 2‐layer flexible PCB panel, consisting of a single layer of polyimide (25 µm) between two layers of copper traces (12.5 µm) and electroplated gold (0.025 µm) which was manufactured externally (PCBWay). The individual functional components of the flexible circuit included the secondary antenna coil, the passive resonator, the main body of the device, the serpentine interconnect, and the electrode array for stimulation. The parts were depaneled using a UV (355 nm) laser ablation system (LPKF; Protolaser U4) and the surface was cleaned using isopropanol (IPA). The antenna, resonator, and electrode were attached to the main device board using low‐temperature solder (Chip Quik; TS391LT) and secured using thermally activated epoxy (Henkel Loctite; 3621 Red Epoxy). The curing of the epoxy was accelerated by heating the device in an oven at 90 °C for 10 min.

### Electrical Components and Antenna Tuning

The secondary antenna coil contained the half‐bridge rectifier circuit, constructed using low forward voltage Schottky diodes (Skyworks Inc.), a 2.2 µF capacitor for smoothing, and a 5.6 V Zener diode (Comchip Technology Corporation) to provide overvoltage protection. Capacitors (82, 7.5, 6, and 4 pF) were used in parallel to tune the secondary antenna and the passive resonator to ensure resonance at the operating frequency of the primary antenna (13.56 MHz). The tuning of the device antenna was matched to 13.56 MHz empirically by testing the devices on a reflection bridge (Siglent; SSA 3032X; RB3×20) and optimization to the lowest voltage standing wave ratio at 13.56 MHz. On the main device body, a 5 V LDO was used to stabilize the input voltage and power the Quad Channel DAC (Maxim Integrated; MAX5713), and the input voltage for the µC (Atmel; ATTINY84A) was stabilized by a 2.8 V LDO. A capacitor bank (3 × 2.2 µF capacitors) was also incorporated to assist in keeping input voltage stable during high drain events. The µC (Atmel; ATTINY84A) was initially programmed on a custom programmer board that was compatible with Arduino IDE and then mounted on the circuit. This provided the ability to control the red indicator µ‐LED with a 5.1 kΩ current limiting resistor vary the channels used for stimulation through the Quad Channel DAC (Maxim Integrated; MAX5713) and set the stimulation voltage. Wireless communication was accomplished via a custom passive protocol.^[^ ^32^, ^33^, ^36^ ^]^ All the electrical components were reflowed using low‐temperature solder (Chip Quik; TS391LT) and a hot air gun at 360 °C.

### Encapsulation

The fully assembled devices were cleaned with isopropanol (IPA) to ensure the surface was free of any debris and solder. The digital device body (excluding the antenna and electrodes) was then covered in thermally activated epoxy (Henkel Loctite; 3621 Red Epoxy) and cured at 90 °C for another 10 min. The layer of epoxy served as a firm anchor for the components when mechanical strain was applied to the device and also provided a uniform layer to help the parylene‐C coating to adhere to the surface of the device. Conformal coating was achieved using parylene encapsulation which was done using the Parylene P6 coating system (Diener Electronic GmbH, Germany), where the devices were hung from a wire and sprayed with two coats of Parylene C dimer. This conformal coating was biocompatible and serves as a moisture barrier in addition to being thin, which allowed the devices to remain flexible. Finally, the entire device body was encapsulated in Ecoflex (Smooth‐On; Silicone), which was a flexible, biodegradable elastomer. This process facilitated mechanical modulus matching to the surrounding tissue minimizing foreign body response and provided mechanical protection against surgical tools during the implantation process.

### Power Characterization

The voltage and power harvesting characteristics of the resonant antenna and the non‐resonant antenna (Figures S1 and S2, Supporting Information) were recorded (Aneng; AN8008) using varying load resistors and a custom device setup with only the resonant antenna (secondary coil and resonator) populated with the necessary components (rectifier circuit and tuning capacitors). The antenna was placed at the center of the 30 cm × 15 cm cage and the RF power was set to 5 W. The load power curve provided an optimal load resistor value which was used to collect voltage and power harvested data (Aneng; AN8008) for varying RF power (0–10 W) for the 30 cm × 15 cm cage. The secondary antenna also tilted from −90° to 90° about its horizontal (pitch) and vertical (roll) axis and the voltage and power harvested values were collected. Additionally, this setup was attached to 3D printed stands (6 and 3 cm heights) and moved throughout the 30 cm × 15 cm cage to record voltage (Aneng; AN8008) and obtain a spatial power harvesting data. The current consumption was also recorded for the device (antenna detached), which was powered using a digital power supply and recorded using a current meter (LowPowerLab; CurrentRanger) and an oscilloscope (Siglent; SDS 1202X‐E).

### Drive Voltage Characterization

Shunt resistors were attached to the electrodes to provide varying loads (3, 4.7, 8.2, 15, 22, and 33 kΩ) to the output. The biphasic pulse was observed on an oscilloscope (Siglent; SDS 1202X‐E) using the current meter (LowPowerLab; CurrentRanger), to show the ability of the devices to maintain stable output current for variable impedances. The devices were programmed wirelessly in the testing cage (30 cm × 15 cm) for different amplitude, pulse width (Figure S4, Supporting Information) and period values of the biphasic pulse and the output through the electrodes was observed on the oscilloscope (Siglent; SDS 1202X‐E) to ensure the ability to provide stable output voltage through the electrodes for different stimulation parameters.

### Electrode Stability Testing

The stability testing for the serpentine electrode was conducted in 0.01 m phosphate buffer saline solution for 120 days using the stimulation parameters for the chronic in vivo studies. Three electrode setups were designed, each with the same pulse width (100 µs) and period (1 ms) for the biphasic pulse but different amplitudes (0.4, 0.6, and 1 V), thus stimulating at a 20% duty cycle. These setups were connected to a fully assembled device and the stimulation parameters were preset onto the microcontroller (Atmel; ATTINY84A) and powered using a battery power supply. This setup was left continuously running for 24 h to mimic chronic stimulation conditions and the electrode integrity was observed every third day using a shunt resistor (100 Ω) and impedance was calculated using current through the shunt and voltage recorded using an oscilloscope (Siglent; SDS 1202X‐E). This procedure was repeated till electrode failure (120 days) with the impedance over time being plotted for all three electrodes and weekly images of the electrodes were captured.

### Accelerated Rate Testing

Long‐term characterization of device functionality was observed by placing three different devices in individual sealed glass containers, with the devices submerged in Dulbecco's PBS solution (14190‐136, Gibco, Life Technologies). These glass jars were placed in 37, 60, and 90 °C ovens, respectively, and the device functionality and stimulation output were measured every third day by powering the devices in the field and observing the stimulation output on the oscilloscope (Siglent; SDS 1202X‐E). The Arrhenius scaling was used to estimate the device lifetime from the data obtained for the 60 and 90 °C devices.

### Vertebrate Animal Subjects

Adult Sprague Dawley rats (200–600 g) were used throughout the study. Animals were kept on a 12‐h dark/light cycle, in a humidity‐ and temperature‐controlled environment with food and water available ad libitum. All procedures were approved by the Institutional Animal Care and Use Committee at Washington University in St. Louis.

### Spared Nerve Injury (SNI) Pain Model

Animals were anesthetized using 2% isoflurane and the spared nerve injury model of neuropathic pain was performed to induce mechanical allodynia as previously published.^[^ ^67^, ^68^ ^]^ Briefly, skin over the left lateral thigh was shaved and sterilized with alternative wipes of betadine and 70% ethanol and then incised. The biceps femoris muscle was bluntly dissected to expose the sciatic nerve. The tibial and peroneal branches were ligated with sterile non‐absorbable suture and transected distally leaving the sural branch intact. The muscle incision was closed with an absorbable suture, and the skin incision was closed with staples. Antibiotic ointment was placed on the incision. Rats were monitored for 4 days and allowed to recover one week before undergoing mechanical sensory testing.

### Wireless SCS Device Implant

Animals that demonstrated stable mechanical hypersensitivity of 20% or more compared to baseline paw withdrawal threshold were implanted with a developed SCS device for stimulation experiment. Prior to surgery, devices were gas sterilized with ethylene oxide (EtO) and allowed to dry overnight. Animals were anesthetized using 2% isoflurane and administered buprenorphine SR (1.0 mg kg^−1^) preoperatively. Prior to making the incision, the back was shaved and sterilized with alternating wipes of betadine and 70% ethanol. After verifying a surgical plane of anesthesia, a midline incision was made near the end of the rib cage and extended caudally toward the hips. The T13 and L1 vertebrae were located, and the superficial muscles were bilaterally cut near the spinous processes of the vertebrae. Musculotendinous attachments were cleared from the T13 and L1 dorsal processes and the L1 dorsal process was removed to provide a path for the device lead to enter the epidural space parallel to the spinal cord. A partial laminectomy slightly wider than the electrode lead was made at the midline of the caudal portion of T13. The device lead was inserted into the epidural space ≈1.5 cm rostral from the caudal end of the T13 vertebrae to target the lumber spinal cord region (Figure 1A). The implant was then gently maneuvered into position to avoid direct pressure on the spinal cord, while preserving the vertebral arch and ensuring minimal tissue disruption (Video S3, Supporting Information). This intraoperative footage also confirmed reliable electrode placement for stimulation at varying motor thresholds, validating that the device can be tuned without imposing mechanical stress on spinal structures. The muscle was sutured closed over the device lead to help secure it in place. Then, the device body was loosely sutured to the muscle to prevent movement on the back while avoiding strain on the antenna. Nylon‐interrupted sutures were used to close the skin, and animals were monitored daily for a minimum of 4 days during recovery.

### Behavioral Testing

Motor function of rats was assessed via gait analysis system for rodents (CatWalk XT, Noldus Information Technology, Netherlands). Rats were habituated to the behavioral room for one hour each day prior to behavioral testing. Two days prior to taking a baseline measurement, rats explored and walked across the catwalk area for 10 min a day. During baseline and post implantation measurements, 4 continuous walks where the animal kept a constant velocity with no rearing were recorded. Motor behavior was reported for two baseline days and two days post‐device implant (day 5) for each rat. The hind paw print area and stance time along with body speed were analyzed to assess the effect of the device implant on the motor function of rats.

Paw withdrawal thresholds were measured via electronic von Frey to assess the mechanical hypersensitivity in rats. On the day of assessment, animals were habituated to the testing arena for 20 min. A single‐blinded experimenter performed all mechanical hypersensitivity testing using an electronic von Frey anesthesiometer with rigid tips (800‐gram range, IITC Life Science Inc.). The force required for the rat to withdraw its paw with brisk withdrawal was recorded and each hindlimb was tested 5–6 times separated by 2 min of rest. The highest and lowest value was excluded, and the paw withdrawal threshold was reported as an average of the remaining 3–4 values.

To compare functional scores between the device implantation and sham surgery groups, a two‐sided unpaired two‐samples t‐test was conducted on post‐operative values for hindpaw surface area, body speed, and hindpaw stance time (Figure 4). Percent changes from pre‐ to post‐operative values were also calculated for each parameter, and unpaired t‐tests were performed to assess differences between the two groups (Figure S10, Supporting Information).

### Supra‐Perception Spinal Cord Stimulation (Conventional LF‐SCS)

These experiments were performed using pulse width modulation tuning protocol. Animals were placed in a small acrylic enclosure with a 3D printed 1 cm × 1 cm grid bottom and allowed to habituate for 20 min before turning on the stimulation. First, the motor threshold (MT) was determined. To visualize a motor response, the stimulation period was set to 500 ms (2 Hz frequency), the amplitude was fixed at 3.3 V, and the biphasic pulse width was adjusted until a muscle contraction was visually identified. The shortest pulse width (10 µs) was first tested and if no muscle response was observed, the duration was increased in 15 µs intervals. Once the MT parameters were identified, the 50 Hz stimulation parameters were set to below MT. The targeted pulse width was 70% MT but, in some animals, the shortest width was between 70% and 100% MT. In all animals, no muscle contractions were observed at 50 Hz. After MT testing, animals were stimulated at 50 Hz for 1 h a day for 3 days. Mechanical hypersensitivity was assessed prior to stimulation onset, 30min, and 60 min into low‐frequency stimulation (Figure S9, Supporting Information). Following the final mechanical hypersensitivity assessment for the day, the stimulation was stopped, and the animals were returned to their home cages.

### Sub‐Perception Spinal Cord Stimulation (HF‐SCS)

These experiments were performed using amplitude modulation tuning protocol. Animals were placed in the same small acrylic enclosure used in the low‐frequency stimulation experiment and allowed to habituate for 20 min prior to MT testing. For MT testing, the period was set to 500 ms (2 Hz), and the pulse width was fixed at 100 µs. The stimulus amplitude was initially set to the lowest setting (0.1 V) to find the minimal amplitude that elicits a muscle contraction. If no muscle response was observed, the amplitude was increased in small intervals (0.1 V) until a contraction was observed. Stimulus amplitude for the 1 kHz experiment was set to 70% MT in all animals except one, where the lowest amplitude setting was between 70% and 100% MT. The pulse width was set to 100 µs and the period was 1 ms in the 1 kHz stimulation experiment. After MT was determined, animals were subjected to mechanical hypersensitivity testing in the small enclosure with a 3D printed grid before 1 kHz stimulation began and 1 h into stimulation. After mechanical hypersensitivity testing was finished, the animals were moved to larger enclosures (11 cm × 11 cm) with bedding, food, water, and a nylon bone for the remainder of the 24‐h stimulation period. The enclosure allowed for animals to rear and move as they would in their home cages. At the end of the 24‐h stimulation period, animals were again moved to the mechanical hypersensitivity testing enclosure and allowed to habituate 20 min before the final paw withdrawal threshold assessment.

### Immunohistochemistry

After the SCS experiment concluded, animals were euthanized and transcardially perfused with 1X PBS (70 011 044, Thermo Fisher Scientific, USA) and fixed with 4% paraformaldehyde (441 244, Millipore Sigma, USA). Spinal cord tissue was extracted, and device placement was confirmed. Spinal cord tissue under the device was cryopreserved and sectioned in series on a cryostat (35 µm thick). One series was employed for immunohistochemistry analysis. The slices were first blocked with 3% BSA (A1470, Millipore Sigma, USA) solution in 1X PBS with 0.3% Triton‐X (T9284, Millipore Sigma, USA) for 1 h. Subsequently, the slices were incubated overnight at 4 °C with the following primary antibodies diluted in 3% BSA dissolved in 1X PBS with 0.3% Triton‐X: GFAP at 1:1000 dilution (60190‐1‐IG, Thermo Fisher Scientific, USA) and Iba1 at 1:1000 dilution (10904‐1‐AP, Thermo Fisher Scientific, USA). The tissues were washed three times with 1X PBS and incubated for one hour with the following secondary antibodies: Alexa flour 488 conjugated goat anti‐mouse (A‐11001, Thermo Fisher Scientific, USA) and Alexa flour 568 conjugated goat anti‐rabbit (A‐11011, Thermo Fisher Scientific, USA), respectively both at 1:1000 dilution. The slices were washed three times with 1X PBS, mounted on a glass slide, and covered with a mounting medium containing DAPI. Fluorescence images were acquired using an epifluorescence microscope (Leica DM6 B, Leica Microsystems Inc., USA), focusing on the dorsal region of the spinal cord (Figure S11, Supporting Information). The total area covered by GFAP and Iba1 stained cells in the dorsal region of the spinal cord was quantified using Fiji software to assess the tissue damage associated with the device implant in the epidural space.

### Statistical Analysis

Statistical analyses were performed to evaluate motor function, pain modulation, and device stability. Motor function parameters, including hind paw print area, body speed, and stance time, were analyzed pre‐ and post‐implantation using paired two‐sample t‐tests within each group and unpaired t‐tests for between‐group comparisons. Percent changes from baseline were also assessed using unpaired t‐tests. Paw withdrawal thresholds in the spared nerve injury (SNI) model were analyzed using repeated‐measures one‐way ANOVA with Tukey's post hoc tests for within‐group comparisons and unpaired t‐tests for inter‐group comparisons between low‐frequency and high‐frequency stimulation paradigms. Electrode impedance stability was characterized by recording impedance values during chronic testing, with trends analyzed to assess long‐term reliability. Data were reported as mean ± SEM, and significance thresholds were defined as *p* < 0.

## Conflict of Interest

R.W.G. and J.A.R. are co‐founders of Neurolux, a company that manufactures wireless optoelectronic devices. The device described here uses related technology but is distinct from the current Neurolux portfolio. All other authors declare no competing interests.

## Author Contributions

A.J.W. and T.B. contributed equally to this work. K.W.M., R.W.G., and P.G. conceived the project and designed the studies. A.B., T.B., and D.M.C. designed and fabricated the device structures. A.B. and T.B. designed and tested the passive resonator system for the 13.56 MHz antenna system. A.B. developed the firmware for the devices. T.B. tested the power harvesting characteristics of different antenna systems. T.B. and P.G. designed the chronic impedance testing of the electrodes and T.B. conducted the electrode stability tests. T.B. characterized encapsulation performance by conducting lifetime functionality testing for the devices under long‐term thermal stress. A.J.W, M.P., and K.W.M. designed the spared nerve injury model and behavioral testing experiments for pre‐clinical testing. D.K.W., J.F., and A.J.W. performed the catwalk and mechanical hypersensitivity testing. A.J.W. and K.W.M. conducted the immunohistochemistry analysis on the implant pre‐ and post‐surgery. A.J.W and R.W.G. analyzed the data for the hypersensitivity testing involving low and high‐frequency stimulation. T.B., A.J.W., and P.G. wrote the manuscript.

## Supporting information



Supporting Information

Supplemental Video 1

Supplemental Video 2

Supplemental Video 3

## Data Availability

The data that support the findings of this study are available from the corresponding author upon reasonable request.
