# Supplementary material for: Chronic, Battery‐Free, Fully Implantable Multimodal Spinal Cord Stimulator for Pain Modulation in Small Animal Models
Source: Adv Sci (Weinh). 2025 Apr 4;12(21):2415963. doi: 10.1002/advs.202415963 (PMC12140358; doi:10.1002/advs.202415963)
Supplement: Supplementary file 1 — Supporting Information [file ADVS-12-2415963-s002.pdf]

ADVANCED SCIENCE
Open Access

Supporting Information

for *Adv. Sci.*, DOI 10.1002/advs.202415963

Chronic, Battery-Free, Fully Implantable Multimodal Spinal Cord Stimulator for Pain Modulation in Small Animal Models

Allie J. Widman, Taron Bashar, Alex Burton, David Marshall Clausen, Prashant Gupta, Drew K. Wolf, Jakayla Folarin-Hines, Maria Payne, John A Rogers, Kathleen W. Meacham, Robert W. Gereau and Philipp Gutruf**

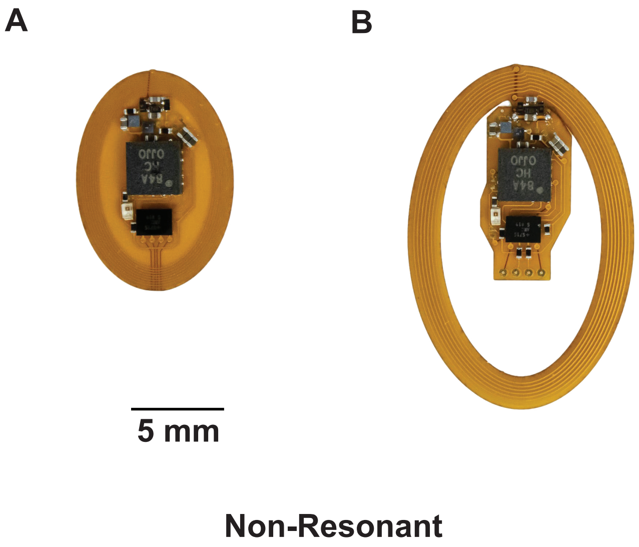

Figure S1. Devices with non-resonant antennas displayed types A and B

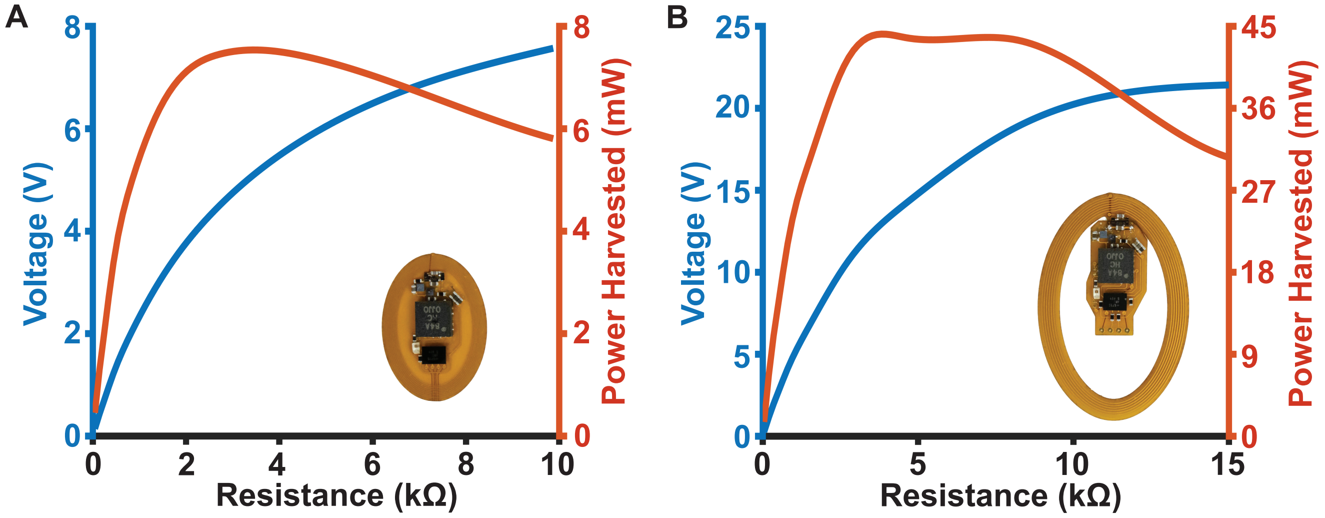

Figure S2. A) Power harvested over varying load displayed for non-resonant antenna type A **B)** Power harvested over varying load displayed for non-resonant antenna type B

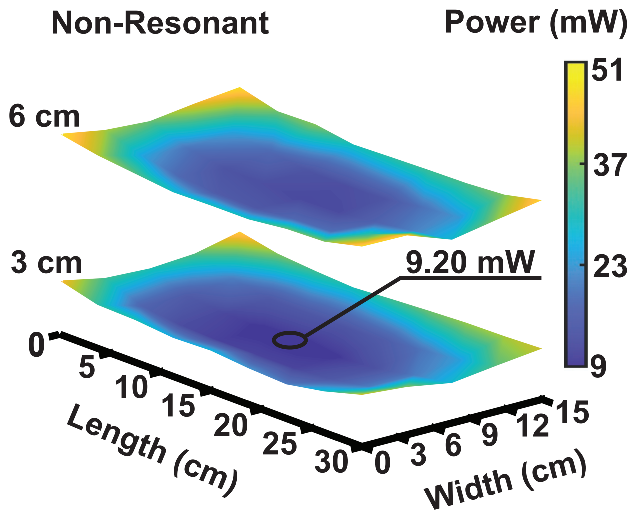

Figure S3. Spatial graph displaying power harvesting capability of non-resonant antenna type A withing testing cage

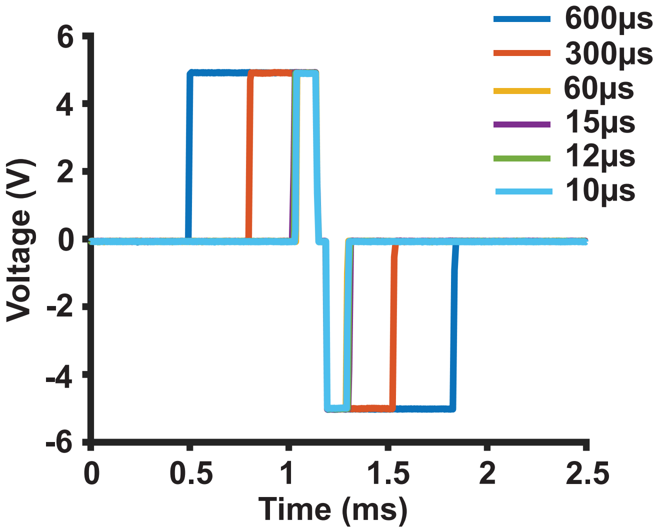

Figure S4. Pulse width modulation capability of the device

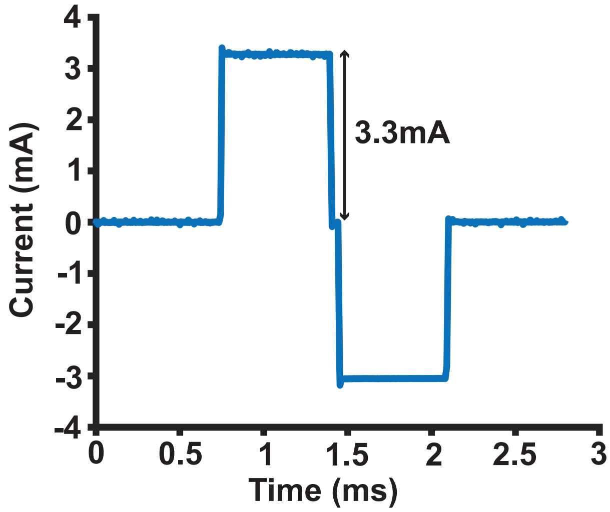

Figure S5. Current delivery through electrodes in PBS solution

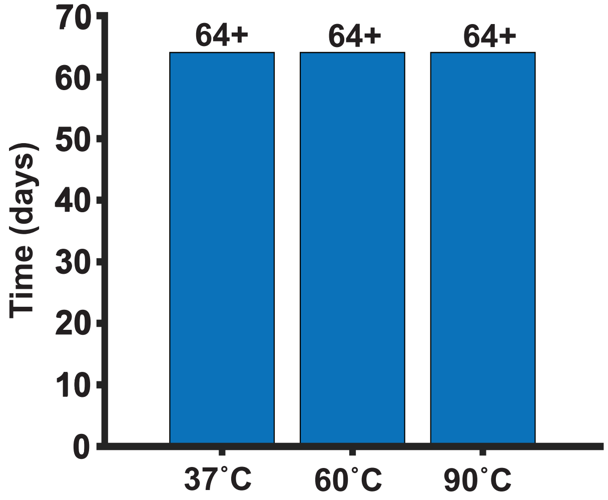

Figure S6. Device operational lifetime while stimulating in PBS at 37˚C, 60˚C and 90˚C

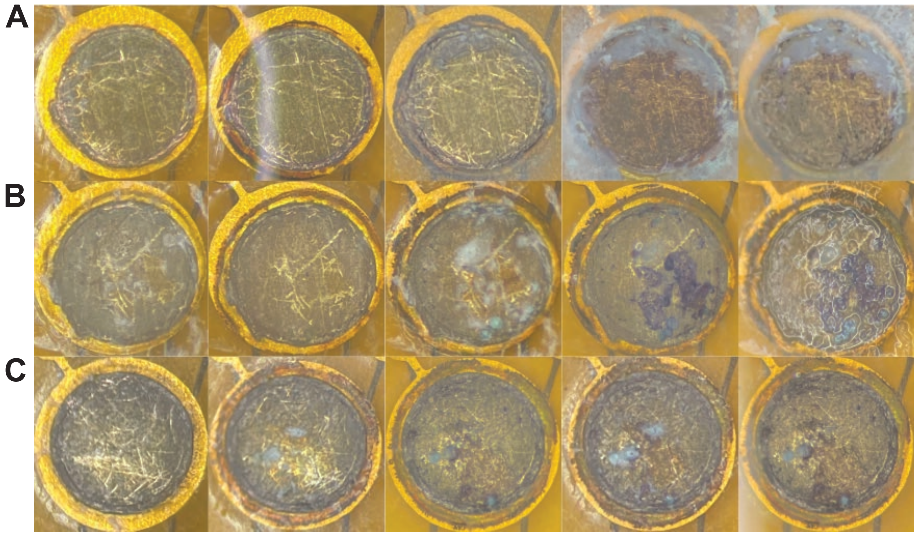

Figure S7. A) Images of electrode degradation at 0.4V stimulation for chronic impedance testing (120 days) **B)** Images of electrode degradation at 0.6V stimulation for chronic impedance testing (120 days) **C)** Images of electrode degradation at 1.0V stimulation for chronic impedance testing (120 days)

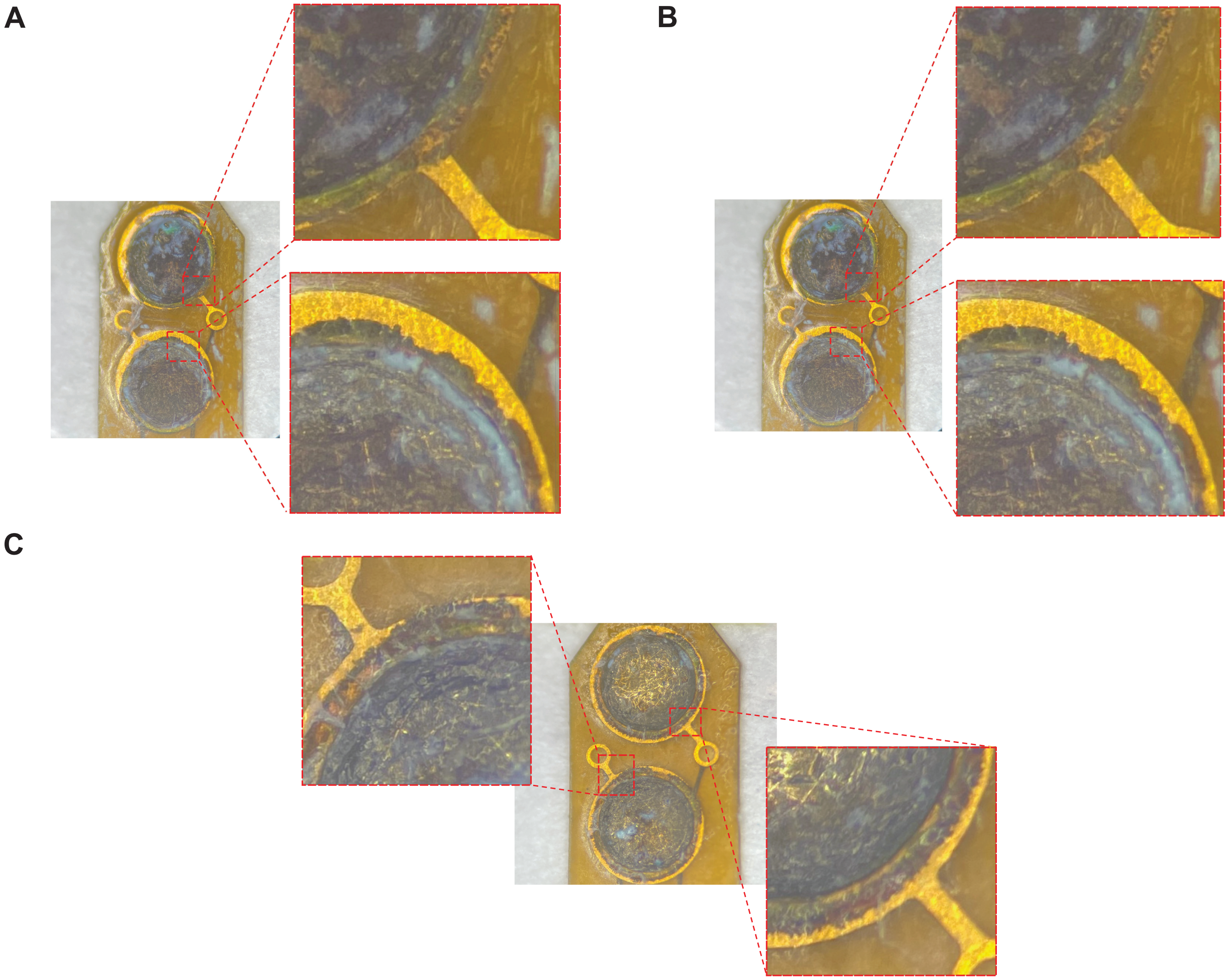

Figure S8. A) Images of electrode running at 0.4V stimulation at Day 120 displaying corrosion sites **B)** Images of electrode running at 0.6V stimulation at Day 120 displaying corrosion sites **C)** Images of electrode running at 1.0V stimulation at Day 120 displaying corrosion sites

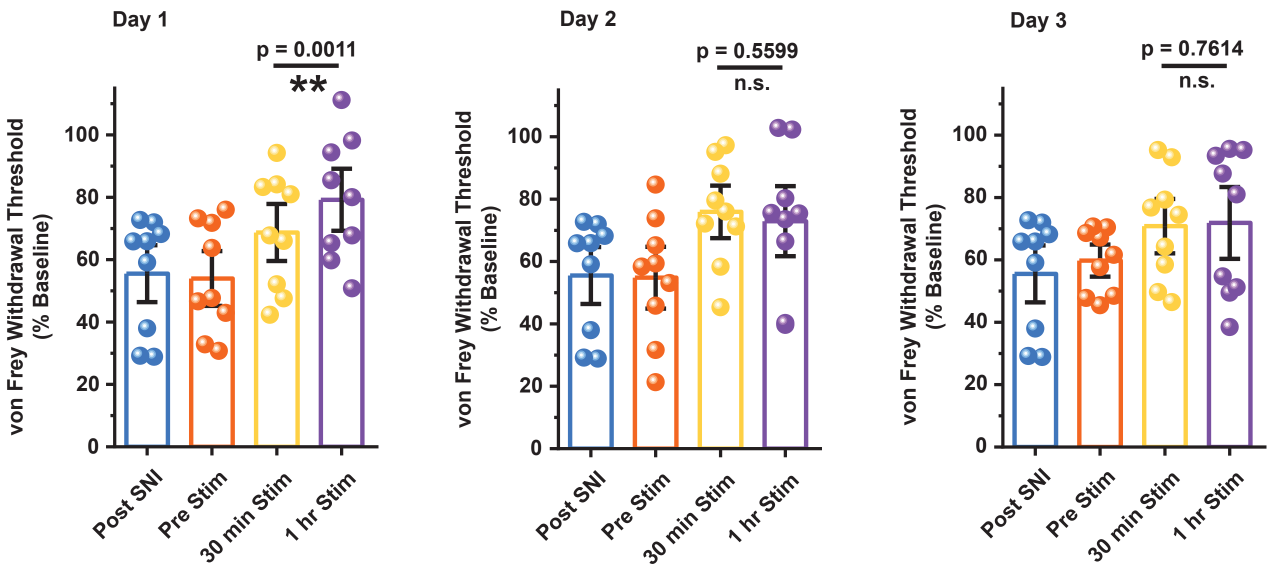

Figure S9. Effect of days on the efficacy of low-frequency spinal cord stimulation (LF-SCS) under an acute stimulation paradigm (30 minutes and 1-hour stimulation) over three consecutive days (n = 9 mice). Data are presented as mean ± S.E.M., with individual data points depicted as filled circles. Statistical analyses were conducted usng repeated measures one-way ANOVA followed by Tukey's post hoc test; n.s. (not significant) $p > 0.05$, * $p < 0.05$, ** $p < 0.01$, *** $p < 0.001$, **** $p < 0.0001$.

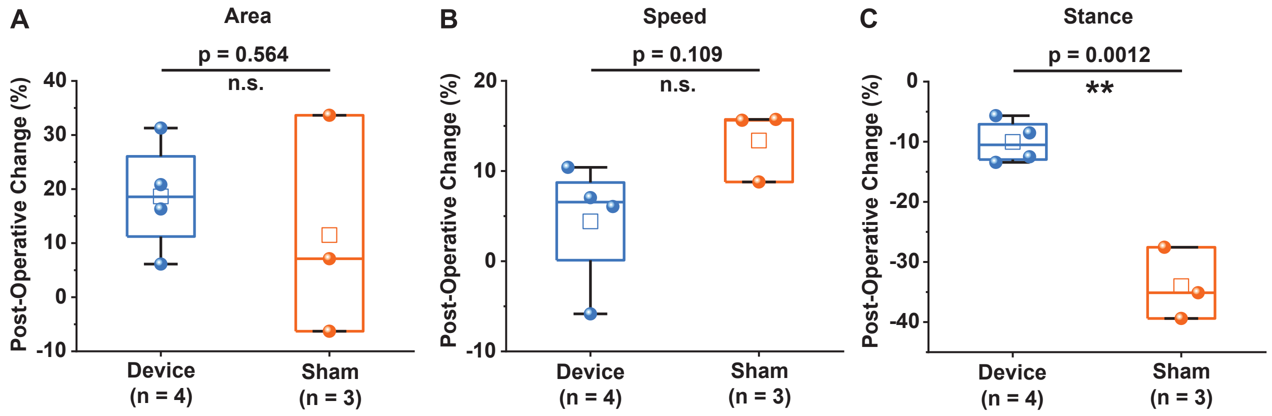

Figure S10. Whisker plots demonstrating the percentage change from pre- to post-operative A) hindpaw surface area, B) body speed and C) hindpaw stance time from device implanted and sham rats (n ≥ 3 rats per group). The box bounds the interquartile range (IQR) divided by the median, and Tukey-style whiskers extend to a maximum of 1.5 x IQR beyond the box. Filled circles are sample data points and open square represents mean. Statistical analyses were performed via two-sided unpaired two-samples t-test; n.s. (not significant) $p > 0.05$, * $p < 0.05$, ** $p < 0.01$, *** $p < 0.001$, **** $p < 0.0001$.

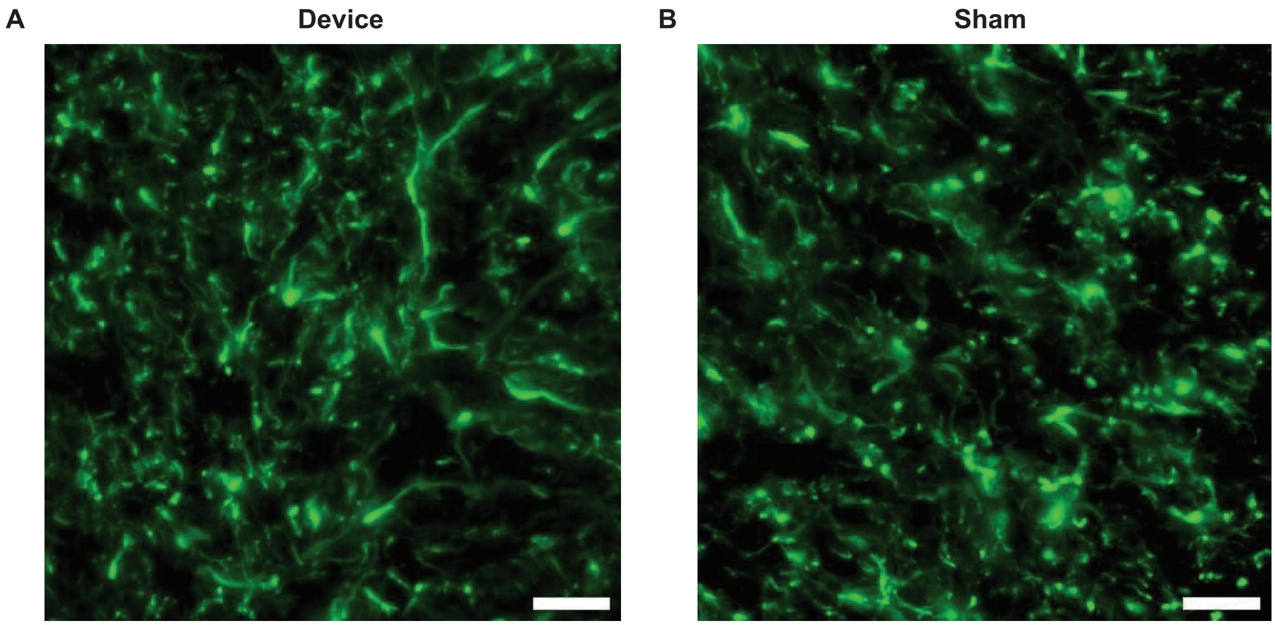

Figure S11. High magnification fluorescence images of transverse spinal cord sections from **A)** device implanted and **B)** sham rats. Spinal cord sections are stained with GFAP (green) which is a specific biomarker for astrocytes.
